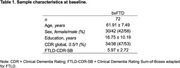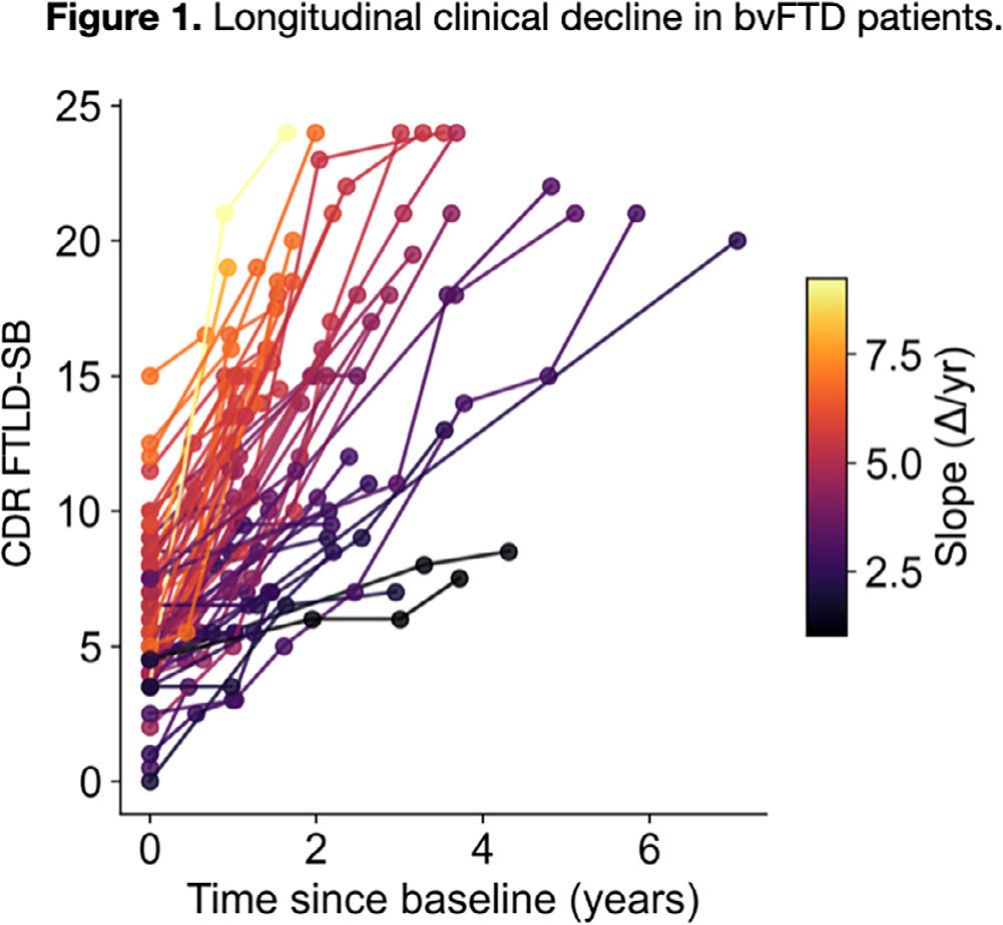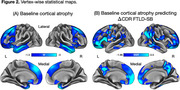# Network‐based cortical atrophy predicts longitudinal clinical decline in sporadic behavioral variant frontotemporal dementia

**DOI:** 10.1002/alz70861_108891

**Published:** 2025-12-23

**Authors:** Erin Anita Krahn, Yuta Katsumi, Thiago Paranhos, Michael Brickhouse, Howard J. Rosen, Alexandra Touroutoglou, Bradford C. Dickerson, Mark C. Eldaief

**Affiliations:** ^1^ Frontotemporal Disorders Unit, Department of Neurology, Massachusetts General Hospital and Harvard Medical School, Boston, MA USA; ^2^ Athinoula A. Martinos Center for Biomedical Imaging, Massachusetts General Hospital, Boston, MA USA; ^3^ Department of Neurology, Memory and Aging Center, University of California San Francisco, San Francisco, CA USA; ^4^ Athinoula A. Martinos Center for Biomedical Imaging, Charlestown, MA USA; ^5^ Massachusetts Alzheimer’s Disease Research Center, Massachusetts General Hospital, Boston, MA USA; ^6^ Department of Psychiatry, Massachusetts General Hospital and Harvard Medical School, Boston, MA USA

## Abstract

**Background:**

Behavioral variant frontotemporal dementia (bvFTD) is a clinically heterogeneous syndrome characterized by progressive deficits across a range of socioemotional and cognitive functions, such as apathy, disinhibition, loss of empathy, impulsivity, and executive dysfunction. Clinical prognostication in bvFTD remains a challenge due to the considerable variability in the rate of clinical progression and symptomatology, and the field currently lacks robust and reliable tools. Here, we investigated the utility of MRI‐based cortical atrophy as a predictor of longitudinal clinical decline in a sample of sporadic bvFTD patients.

**Method:**

We analyzed data obtained from two independent cohorts of bvFTD patients: the Massachusetts General Hospital cohort (n = 39) and the FTLD‐NI cohort (n = 33). All patients had cortical thickness estimates from baseline MRI scans, which were used to predict longitudinal change in clinical impairment assessed by the CDR Sum‐of‐Boxes score adapted for FTLD (CDR FTLD‐SB). Multivariate partial least squares (PLS) analysis was performed to identify a weighted linear combination of cortical vertices where the magnitude of atrophy is maximally associated with the rate of CDR FTLD‐SB change across bvFTD patients.

**Result:**

The current sample included 72 patients with sporadic bvFTD (Table 1). Each patient had at least one clinical follow‐up, with the mean duration of 2.00 ± 1.28 years. On average, CDR FTLD‐SB scores increased by 4.40 points annually (p < .0001) (Figure 1). bvFTD patients exhibited prominent cortical atrophy at baseline in anterior cortical regions, including those part of the limbic network (e.g., temporal pole) as well as the anterior nodes of the default mode network (e.g., dorsomedial prefrontal cortex, rostral lateral temporal cortex) (Figure 2A) Multivariate vertex‐wise PLS analysis showed that default mode network regions spatially distinct from those exhibiting baseline atrophy (e.g., ventromedial prefrontal cortex, posteromedial cortex, inferior parietal lobule) most prominently and reliably predicted the rate of subsequent clinical decline (Figure 2B).

**Conclusion:**

These results point to the clinical prognostic utility of MRI‐derived cortical atrophy in sporadic bvFTD. We propose a *latent network vulnerability* model of clinical progression in neurodegenerative disease, which may provide a unified framework for estimating person‐specific trajectories of clinical decline and optimizing clinical trial designs.